# Association of vitamin A and D deficiency and the presence of sepsis in the geriatric population: a cross-sectional study

**DOI:** 10.3389/fnut.2025.1676174

**Published:** 2025-11-12

**Authors:** Yichuan Feng, Pengfei Xuan, Ping Kang, Jingping Yang, Hongyan Wang, Tiewei Li

**Affiliations:** 1 Department of Clinical Laboratory, Zhengzhou Key Laboratory of Children’s Infection and Immunity, Henan Children’s Hospital, Children’s Hospital Affiliated to Zhengzhou University, Zhengzhou, Henan, China; 2 Respiratory and Critical Care Medicine Department, Inner Mongolia Baogang Hospital, Baotou, China

**Keywords:** sepsis, vitamin A, vitamin D, deficiency, geriatric population

## Abstract

**Background:**

Extensive research has established that vitamins A (VA) and D (VD) are essential to immune function. Deficiencies in these vitamins are associated with increased susceptibility to infections and more severe disease outcomes. However, the relationship between VA and VD deficiency and sepsis in geriatric persons (aged > 60 years) remains underexplored. The aim of this study was to investigate the association between sepsis incidence in persons over 60 and deficiencies in VA and VD.

**Methods:**

39 geriatric patients diagnosed with sepsis between August 2024 and April 2025 were consecutively enrolled. Among the sepsis patients, 15 succumbed during hospitalization. During the same period, 28 geriatric patients hospitalized with common infectious diseases were recruited as controls. Online medical files at the time of hospitalization were used to gather medical and laboratory information retrospectively. Everyone who participated had their peripheral blood samples taken, and ultra-performance liquid chromatography tandem mass spectrometry helped us assess serum concentrations of 25-hydroxyvitamin D3 [25(OH)D3], 25-hydroxyvitamin D2 [25(OH)D2], and retinol (VA). The combined concentration of 25(OH)D3 and 25(OH)D2 helped calculate the overall VD levels. SPSS 24.0 (IBM Corp., Armonk, NY, USA) helped carry out all analyses.

**Results:**

In comparison to controls, geriatric patients with sepsis demonstrated significantly lower serum VA and VD levels, alongside a notably higher deficiency rate for both vitamins. Correlation analyses revealed significant inverse associations between serum levels of VA and VD and the infection marker procalcitonin (PCT) as well as the inflammatory marker interleukin-6 (IL-6). Multivariate regression analysis showed that in persons over 60, deficiencies in either VA or VD were independently associated with significantly higher odds of sepsis.

**Conclusion:**

Vitamins A and VD deficiencies were associated with lower serum levels in geriatric sepsis patients and were inversely correlated with PCT and IL-6. Furthermore, deficiencies in either vitamin were independently associated with a higher prevalence of sepsis in this population.

## Introduction

Sepsis is a potentially fatal condition characterized by organ malfunction or failure resulting from a dysregulated systemic inflammatory response to infection (1). This syndrome poses a particularly high risk to geriatric patients, significantly contributing to morbidity and mortality in this demographic (2). Unlike younger adults, the elderly experience age-related immunosenescence, characterized by impaired neutrophil phagocytosis, reduced T-cell diversity, and persistent low-grade inflammation, which render them more susceptible to severe infections that progress to sepsis and fatal outcomes (3–5). Despite advances in antimicrobial therapies and organ support, geriatric patients face a markedly higher risk of sepsis, with increased disease severity and mortality rates compared to younger adults (6–8). This heightened risk persists even after adjusting for comorbidities, highlighting the urgent need to identify modifiable factors specific to elderly pathophysiology.

Vitamins play essential roles in maintaining physiological homeostasis (9). Accumulating evidence highlights the critical roles of vitamins A (VA) and D (VD) in immune function: both vitamins regulate inflammatory cytokine release and assist in controlling excessive inflammation, with clinical studies consistently showing an inverse relationship between their serum levels and disease severity (10–13). Older adults commonly exhibit lower circulating levels of VA and VD than younger populations (14, 15), a pattern we also observed in our previous study of elderly COVID-19 patients (16). Despite this, the association between VA and VD status and sepsis in acutely ill older adults remains inadequately explored. Therefore, this study was designed to evaluate whether deficiencies in these vitamins are associated with sepsis incidence and clinical severity in adults over 60 years of age.

## Materials and methods

### Study design

This cross-sectional study was conducted between August 2024 and April 2025. A total of 39 geriatric patients (aged > 60 years) who met the Sepsis-3 diagnostic criteria were consecutively enrolled into the sepsis group. During the same period, 28 control patients with common infectious diseases not meeting sepsis criteria were also recruited. Sepsis diagnosis was validated according to Sepsis-3 criteria, and sequential organ failure assessment (SOFA) scores were used to assess organ dysfunction severity. Inclusion criteria for the sepsis group comprised: (1) age > 60 years and (2) fulfillment of Sepsis-3 clinical diagnostic criteria. The control group consisted of patients over 60 with confirmed infections but not meeting sepsis thresholds. Both groups shared the following exclusion criteria: (1) incomplete clinical or laboratory data; (2) history of autoimmune diseases, hematological disorders, or concurrent malignancies; and (3) inability or unwillingness to provide written informed consent. As this study employed a consecutive enrollment design without prior sample size calculation, all eligible patients during the study period were included. The Inner Mongolia Baogang Hospital’s Medical Ethics Committee authorized the research procedure (Approval No: 2022-MER-110), and it complied with the Declaration of Helsinki. Written informed consent was obtained from all participants prior to enrollment.

### VA and VD detection

1. Serum Collecting and Processing: On day 1, venous blood samples (4 mL) were collected into plain tubes without anticoagulants. After clotting for 30 min, the samples were centrifuged at 500 × *g* for 10 min. The serum layer was then carefully separated, aliquoted, and stored at −80 °C for subsequent analysis.
2. Measurement of VA and VD: Serum retinol (VA) and 25-hydroxyvitamin D2/D3 levels were measured using a Waters liquid chromatography-tandem mass spectrometry system (Waters Corporation, MA, USA). Samples were prepared using commercial vitamin assay kits from Tianjin Huahao Biotechnology Co., Ltd., (China) according to the manufacturer’s instructions. A 5-μL aliquot was injected for analysis using electrospray ionization in positive ion mode with the following parameters: desolvation gas flow 1,000 L/h, cone gas flow 50 L/h, ion source temperature 150 °C, capillary voltage 3.0 kV, and desolvation temperature 500 °C. Data were acquired and processed using MassLynx™ software (Waters Corporation, Milford, MA, USA). Vitamin A and D deficiency were defined according to Chinese national standards, which are aligned with prevalent international definitions. Specifically, per the Chinese Standard WS/T 553-2017, VA deficiency was defined as serum retinol <200 ng/mL (<0.70 μmol/L) (17), a cut-off that is consistent with the World Health Organization (WHO) international standard-(18). Per the Screening Methods for Vitamin D Deficiency in China (WS/T 677-2020), which also aligns with international definitions (e.g., Mayo Clinic Laboratories), vitamin D deficiency is defined as serum 25-hydroxyvitamin D <20 ng/mL (19).
3. Validation: The analytical method was rigorously validated for linearity, precision, accuracy, and limit of quantification (LOQ) for VA, VD2, and VD3.
  - Linearity: The calibration curves demonstrated excellent linearity over the following concentration ranges: 10–2,000 ng/mL for VA, 0.25–50 ng/mL for vitamin D2, and 0.5–100 ng/mL for vitamin D3. The correlation coefficients (r) for all compounds ranged from 0.996 to 0.998, meeting the pre-defined acceptance criterion of *r* > 0.990.
  - Precision: Intra-day and inter-day precision were evaluated at two concentration levels for each analyte over three consecutive days. For VA, quality control (QC) levels were 150 and 1,500 ng/mL; for vitamin D2, 3.3 and 33 ng/mL; and for vitamin D3, 6.6 and 66 ng/mL. The maximum observed intra-day and inter-day coefficients of variation (CV) were 5.07% and 6.56%, respectively, well within the acceptable limit of ≤ 15%.
  - Accuracy (Recovery): Accuracy was assessed via spike-recovery experiments using human serum samples at the aforementioned QC levels. The recovery rates for VA, VD2, and VD3 ranged from 89.93% to 110.3%, all conforming to the acceptable range of 85%–115%.
  - Limit of Quantification (LOQ): The LOQ was established as the lowest concentration on the calibration curve that could be measured with acceptable precision and accuracy. The LOQs were defined as 10 ng/mL for VA, 0.25 ng/mL for vitamin D2, and 0.5 ng/mL for vitamin D3. At these levels, the method demonstrated a maximum CV of 7.70% and concentration deviations within −5.35% to 2.26%, satisfying the acceptance criteria of ±15% for deviation and ≤20% for CV.
  - Quality control (QC) samples analyzed alongside clinical specimens consistently remained within ±10% of their nominal values throughout the study.

### Data collection

At the time of hospitalization, demographic information, laboratory results, and clinical traits were extracted from online medical files. The following clinical variables were considered: demographics (age, sex, body mass index); vital signs (body temperature, respiratory rate, heart rate, systolic and diastolic blood pressure); laboratory parameters [platelet count (PLT), neutrophil count, blood urea nitrogen (BUN), creatinine (CREA), hemoglobin (HGB), alanine aminotransferase (ALT), aspartate aminotransferase (AST)]; comorbidities (history of diabetes mellitus, hypertension, heart disease); and disease severity markers SOFA score, (procalcitonin (PCT), and interleukin-6 (IL-6) levels). Siemens Healthineers’s Siemens Advia 2,400 automated biochemistry analyzer (Erlangen, Germany) helped measure serum AST, CREA, BUN, ALT levels. Maccura Biotechnology’s Maccura F81 automated hematology analyzer (Sichuan, China) helped find HGB, PLT and neutrophil counts. Roche Diagnostics’ Cobas 8,000 modular analyzer platform (Rotkreuz, Switzerland) helped carry out an electrochemiluminescence immunoassay to find IL-6 and PCT concentrations. PCT values exceeding the upper measurable limit (>100 ng/mL) were recorded as 101 ng/mL.

### Statistical analysis

SPSS software 26.0 (IBM Corp., Armonk, NY, USA) helped analyze all information. The mean ± standard deviation shows continuous variables that were normally distributed, and independent samples *t*-tests helped compare them. The median with interquartile range shows variables that were not distributed normally, and the Mann-Whitney U test helped compare them. Frequencies and percentages show categorical variables, and the chi-square test helped compare them. The Spearman’s rank correlation coefficient helped find relationships between continuous variables. Binary logistic regression using the enter method was employed to identify independent factors associated with sepsis. In the multivariate logistic regression model, indicators that had *P* < 0.05 in the univariate logistic regression were added as covariates. *P* < 0.05 (two-tailed) meant significance.

## Results

### Study population characteristics

This research included 39 geriatric patients diagnosed with sepsis and 28 patients with general infections, all aged more than 60. The Sepsis group contained people who met the sepsis diagnostic standards, whereas the Control group contained people with infections but not diagnosed with sepsis. In the sepsis group, 15 experienced in-hospital mortality. The baseline traits are detailed in Table 1. Compared to the Control group, the Sepsis group exhibited significantly higher body temperature, respiratory rate, AST, BUN, neutrophil counts, PCT, and SOFA scores (*P* < 0.05), along with significantly lower PLT counts, SBP, DBP, and serum levels of VA and VD (*P* < 0.05). No significant differences were observed in BMI, HGB, or the prevalence of diabetes mellitus, hypertension, or heart disease between the two groups (*P* > 0.05). Within the Sepsis group, non-survivors had significantly higher heart rate, BUN levels, and SOFA scores than survivors (*P* < 0.05). However, no other clinical characteristics, including ALB levels, differed significantly between survivors and non-survivors (*P* > 0.05).

**TABLE 1:** Basic characteristics of study subjects by groups.

Variables	Control (*n* = 28)	Sepsis (*n* = 39)	P^a^	Sepsis		
				Survivors (*n* = 24)	Non-survivors (*n* = 15)	P^b^
Age (years)	77.4 ± 9.4	78.7 ± 7.6	0.546	78.1 ± 7.5	79.6 ± 8.0	0.566
Male, *n* (%)	15 (53.6%)	22 (56.4%)	0.818	13 (54.2%)	9 (60.0%)	0.721
BMI (kg/m^2^)	22.6 (19.7, 25.2)	21.2 (16.7, 24.5)	0.176	21.9 (18.8, 25.7)	19.5 (15.4, 23.2)	0.131
Temperature (°C)	36.8 ± 0.8	37.5 ± 1.0	**0.007**	37.4 ± 0.6	37.5 ± 1.1	0.783
Respiratory (rate/minute)	20.8 ± 0.7	22.8 ± 3.6	**0.001**	23.3 ± 4.0	22.0 ± 2.6	0.278
Heart rate (rate/minute)	87.5 (78.2, 110.7)	98.0 (84.0, 110.0)	0.213	89.5 (80.2, 100.0)	110.0 (104.0, 122.0)	**<0.001**
SBP (mm Hg)	138.5 (125.3, 150.5)	115.0 (95.0, 130.0)	**<0.001**	118.0 (101.2, 131.5)	110.0 (87.0, 127.0)	0.309
DBP (mm Hg)	81.5 (67.0, 86.5)	71.0 (60.0, 78.0)	**0.006**	71.0 (60.0, 77.8)	66.0 (60.0, 89.0)	0.765
Hypertension, *n* (%)	12 (42.9%)	25 (64.1%)	0.085	14 (58.3%)	11 (73.3%)	0.342
Diabetes, *n* (%)	22 (78.6%)	32 (82.1%)	0.722	21 (87.5%)	11 (73.3%)	0.262
Heart disease, *n* (%)	20 (71.4%)	30 (76.9%)	0.61	19 (79.2%)	11 (73.3%)	0.674
**Biochemical parameters**						
ALT (U/L)	15.5 (10.5, 20.0)	17.0 (**??**)	0.434	17.0 (11.2, 25.5)	18.0 (6.0, 72.0)	0.921
AST (U/L)	20.5 (15.2, 29.0)	29.0 (18.0, 49.0)	**0.017**	26.5 (18.2, 43.7)	32.0 (18.0, 71.0)	0.578
BUN (nmol/L)	5.9 (5.0, 7.2)	10.3 (5.9, 16.5)	**0.002**	7.4 (5.1, 12.7)	14.0 (10.3, 31.7)	**0.01**
CREA (μmol/L)	68.3 (52.7, 90.6)	93.7 (54.2, 145.3)	0.075	89.3 (56.2, 117.6)	109.6 (53.3, 279.0)	0.246
HGB (g/L)	126.0 (113.5, 143.0)	128.0 (99.0, 141.0)	0.472	126.0 (110.0, 140.3)	132.0 (99.0, 143.0)	0.966
Neutrophil (10^9^ cells/L)	5.12 (3.71, 8.27)	8.24 (5.18, 13.75)	**0.026**	6.86 (4.51, 12.26)	10.92 (5.18, 20.23)	0.296
PLT (10^9^ cells/L)	204.0 (170.5, 239.0)	137.0 (92.0, 245.0)	**0.014**	143.0 (101.0, 211.7)	120.0 (90.0, 300.0)	0.943
PCT (ng/mL)	0.20 (0.07, 0.70)	1.97 (0.55, 6.16)	**<0.001**	1.68 (0.45, 19.85)	2.07 (0.55, 4.77)	0.921
IL-6 (pg/mL)	84.5 (14.0, 253.7)	118.4 (66.8, 550.9)	**0.037**	86.5 (21.8, 403.7)	196.6 (99.6, 901.8)	0.071
VA (ng/mL)	241.8 (173.3, 337.8)	165.9 (112.4, 327.9)	**0.017**	151.5 (117.6, 308.5)	179.8 (55.9, 337.6)	0.966
VD (ng/mL)	13.0 (8.9, 21.4)	8.8 (4.6, 13.4)	**0.002**	8.1 (4.9, 11.2)	9.4 (3.1, 19.4)	0.765
SOFA	1.0 (0, 2.0)	5.0 (3.0, 8.0)	**<0.001**	3.5 (3.0, 6.75)	7.0 (5.0, 11.0)	**0.005**

Boldface highlights results with statistically significant differences.

P^a^ value among the control and sepsis groups.

P^b^ value among the survivor, and non-survivor sepsis groups. ALT, alanine aminotransferase; AST, aspartate aminotransferase; BMI, body mass index; BUN, blood urea nitrogen; CREA, creatinine; HGB, hemoglobin; IL-6, interleukin-6; PCT, procalcitonin; PLT, platelet; SOFA, sequential organ failure assessment; VA, vitamin A; VD, vitamin D.

### VA and VD nutritional status

In accordance with the Chinese industry standards WS/T 677-2020 as well as WS/T 553-2017, VA deficiency was defined as a serum retinol concentration <200 ng/mL, with concentrations =200 ng/mL considered sufficient. Similarly, serum VD levels <20 ng/mL meant VD deficiency, with =20 ng/mL regarded as sufficient. In comparison to the control group, the data revealed a significantly higher prevalence of VA deficiency in the sepsis group (32.1% vs. 64.1%, *P* = 0.010). Likewise, the control group showed a lower prevalence of VD deficiency than the sepsis group (64.3% vs. 89.7%, *P* = 0.011). Furthermore, VA or VD deficiency did not differ between sepsis non-survivors and survivors (*P* > 0.05) (Table 2).

**TABLE 2:** The nutritional status of VA and VD in the control and sepsis groups.

Variables	Control (*n* = 28)	Sepsis (*n* = 39)	P^a^	Sepsis		
				Survivors (*n* = 24)	Non-survivors (*n* = 15)	P^b^
VA status			**0.010**			0.673
VA sufficiency, *n* (%)	19 (67.9%)	14 (35.9%)		8 (33.3%)	6 (40.0%)	
VA deficiency, *n* (%)	9 (32.1%)	25 (64.1%)		16 (66.7%)	9 (60.0%)	
VD status			**0.011**			
VD sufficiency, *n* (%)	10 (35.7%)	4 (10.3%)		1 (4.2%)	3 (20.0%)	0.113
VD deficiency, *n* (%)	18 (64.3%)	35 (89.7%)		23 (95.8%)	12 (80.0%)	

Boldface highlights results with statistically significant differences.

P^a^ value among the control and sepsis groups.

P^b^ value among the survivor and non-survivor sepsis groups. VA, vitamin A; VD, vitamin D.

### Correlation of VA and VD with clinical indicators

Correlation analysis explored the relationships among clinical parameters. As shown in Table 3, VA levels were negatively related to the heart rate, PCT and IL-6 (*P* < 0.05). However, VA was not related to age, temperature, respiratory rate, SBP, DBP, ALT, AST, BUN, CREA, neutrophil count, PLT, or SOFA scores (*P* > 0.05). VD levels were negatively related to the respiratory rate and PCT (*P* < 0.01), but did not to age, temperature, heart rate, SBP, DBP, AST, BUN, ALT, PLT, CREA, neutrophil count, or SOFA scores (*P* > 0.05).

**TABLE 3:** Correlation between VA, VD and clinical indexes.

Variables	VA		VD	
	*r*	*P*	*r*	*P*
Age (year)	−0.066	0.594	−0.08	0.518
Temperature (°C)	−0.182	0.141	−0.201	0.104
Respiratory (rate/minute)	−0.170	0.170	**−0.316**	**0.009**
Heart rate (rate/minute)	−0.265	**0.030**	−0.123	0.322
SBP (mm Hg)	0.136	0.274	0.236	0.054
DBP (mm Hg)	0.200	0.104	0.185	0.133
ALT (U/L)	0.053	0.668	−0.024	0.848
AST (U/L)	0.015	0.907	−0.149	0.228
BUN (nmol/L)	−0.003	0.981	−0.193	0.117
CREA (μmol/L)	0.053	0.672	−0.099	0.428
Neutrophil (×10^9^ cells/L)	−0.220	0.073	−0142	0.253
PLT (×10^9^ cells/L)	0.079	0.527	0.163	0.188
PCT (ng/mL)	−0.381	**0.001**	**−0.410**	**0.001**
IL-6 (pg/mL)	−0.402	**0.001**	−0.183	0.142
SOFA	−0.168	0.175	−0.23	0.061

Boldface highlights results with statistically significant differences. ALT, alanine aminotransferase; AST, aspartate; BUN, blood urea nitrogen; CREA, creatinine; IL-6, interleukin-6; PCT, procalcitonin; PLT, platelet; SOFA, sequential organ failure assessment; VA, vitamin A; VD, vitamin D.

### Association of VA and VD status with sepsis

To examine whether VA and VD are independent factors associated with sepsis in older adults, multivariable binary logistic regression analysis was performed. The adjusted model contained variables (neutrophil count, body temperature, SBP, AST, and respiratory rate) that showed significant associations (*P* < 0.05) in univariate regression. As shown in Table 4, in the unadjusted analysis (Model 1), deficiencies of both VA and VD were associated with higher odds of sepsis. These associations remained significant after sequential adjustment for body temperature, respiratory rate, and SBP (Model 2), and further for AST and neutrophil counts (Model 3). In the fully adjusted model (Model 3), geriatric patients with VA deficiency were associated with 6.3-times higher odds (OR = 6.297, 95% CI = 1.187–33.402, *P* = 0.031), and those with vitamin D deficiency were associated with 11.4-times higher odds (OR = 11.386, 95% CI = 1.199–108.097, *P* = 0.034) of developing sepsis compared to individuals with adequate vitamin levels.

**TABLE 4:** Logistic regression analysis of the association between VA/VD deficiency and sepsis.

Model	Variables	β	OR (95% CI)	*P*	Nagelkerke *R*^2^	Hosmer-lemeshow test (*P*)
Model 1	VA (ng/mL)	−0.003	0.997 (0.993–1.000)	0.071	0.068	0.122
	VA status					
	VA sufficiency		1			
	VA deficiency	1.327	3.770 (1.348–10.540)	**0.011**	0.13	–
	VD (ng/mL)	−0.097	0.908 (0.846–0.974)	**0.007**	0.182	0.662
	VD status					
	VD sufficiency		1			
	VD deficiency	1.581	4.861 (1.336–17.684)	**0.016**	0.122	–
Model 2	VA (ng/mL)	−0.002	0.998 (0.993–1.003)	0.365	0.508	0.298
	VA status					
	VA sufficiency		1			
	VA deficiency	1.295	3.652 (0.976–13.663)	**0.049**	0.549	0.892
	VD (ng/mL)	−0.074	0.928 (0.850–1.014)	0.098	0.539	0.135
	VD status					
	VD sufficiency		1			
	VD deficiency	2.036	7.662 (1.292–45.442)	**0.016**	0.57	0.447
Model 3	VA (ng/mL)	−0.004	0.996 (0.989–1.003)	0.259	0.634	0.306
	VA status					
	VA sufficiency		1		1	
	VA deficiency	1.840	6.297 (1.187–33.402)	**0.031**	0.676	0.259
	VD (ng/ml)	−0.104	0.902 (0.806–1.009)	0.071	0.664	0.193
	VD status					
	VD sufficiency		1			
	VD deficiency	2.432	11.386 (1.199–108.097)	**0.034**	0.678	0.058

Boldface highlights results with statistically significant differences. Model 1: unadjusted; Model 2: adjusted for body temperature, respiratory rate and SBP; Model 3: body temperature, respiratory rate, SBP, AST and neutrophil counts. AST, aspartate aminotransferase; SBP, systolic blood pressure; VA, vitamin A; VD, vitamin D.

## Discussion

Sepsis is a life-threatening condition characterized by a dysregulated host response to infection, leading to organ dysfunction and high mortality (20). The World Health Organization estimates that sepsis affects millions globally each year, contributing significantly to morbidity and placing a substantial economic burden on healthcare systems (21). The geriatric population is particularly vulnerable to sepsis due to age-related immunosenescence and a higher prevalence of comorbidities (22). While the roles of VA and VD in immunity are well-documented, their combined status and association with sepsis in the elderly remain underexplored. This study provides new evidence that deficiencies in both VA and VD are common in geriatric septic patients and constitute independent factors associated with sepsis.

Regarding VA, our findings confirm significantly lower levels in geriatric septic patients compared to non-septic controls. This extends our previous observation in elderly COVID-19 patients and suggests a more pronounced deficit in sepsis (16). The VA deficiency rate in our cohort was also higher than that reported in pediatric sepsis (23), highlighting an age-specific vulnerability. The mechanisms underlying VA’s role are multifaceted. VA is a vital precursor for mucin synthesis, crucial for maintaining mucosal barrier integrity in respiratory, digestive, and urogenital tracts (24). Its deficiency impairs barrier repair and weakens the function of neutrophils, natural killer cells, and macrophages (25). Additionally, its metabolite, retinoic acid, promotes the differentiation of regulatory T cells, which help control excessive inflammation (26). The acute decline in serum retinol during sepsis can be attributed to low baseline status, impaired absorption, and acute-phase response-mediated redistribution (27). Critically, our data provide clinical support for these mechanisms by demonstrating significant negative correlations between VA levels and both IL-6 and PCT, directly linking VA status to inflammation and infection severity in our cohort.

For VD, we similarly observed markedly reduced levels in geriatric septic patients, consistent with previous reports (28–31). The immunomodulatory mechanisms of VD are well-documented: it is converted to active calcitriol in monocytes, enhancing antimicrobial peptide secretion (e.g., cathelicidin) and directly eliminating pathogens (32, 33). Simultaneously, it reduces inflammation by inhibiting proinflammatory cytokines and promoting interleukin-10 synthesis (34), while also strengthening the intestinal epithelial barrier (35). A key advance from our study is that VD deficiency remained independently associated with sepsis after multivariate adjustment, a finding that highlights its potential value in the clinical assessment of geriatric sepsis. Our results align with studies showing lower VD levels in elderly versus younger septic populations (36, 37), reinforcing age-related vulnerability.

The major novel contribution of this study is the integrated assessment of VA and VD status, revealing their independent associations with the prevalence of sepsis in a geriatric cohort. Most prior studies have focused on single vitamins or mixed-age populations, whereas our findings highlight a high burden of VA or VD deficiencies in elderly septic patients - a phenomenon not commonly reported in previous geriatric-focused sepsis literature. The distinct negative correlations of VA with IL-6 and PCT, and of VD with PCT, provide clinical support for the involvement of these vitamins in modulating infection and inflammatory responses in the elderly.

This study has several limitations. Its single-center design and modest sample size limit generalizability, warranting validation in larger, multi-center cohorts. The cross-sectional nature precludes causal inference about whether deficiencies predispose to sepsis or result from it. Furthermore, we lacked data on potential confounders like nutritional intake, sunlight exposure, and socioeconomic status. The absence of longitudinal data also limits assessment of prognostic value. Given these limitations, we caution against overstating the clinical implications. While our findings robustly link VA and VD status to the prevalence of sepsis in the elderly, they do not yet justify routine screening or supplementation. Future prospective studies and randomized trials are essential to determine whether correcting these deficiencies can reduce sepsis incidence or improve outcomes in geriatric populations.

## Conclusion

In conclusion, this cross-sectional study demonstrates that geriatric patients with sepsis exhibit significantly lower serum concentrations and higher deficiency rates of VA and VD compared to non-septic infected controls. The multivariate analysis identified these deficiencies as factors independently associated with the presence of sepsis in this population. These findings highlight a clinically relevant association and suggest that assessing VA and VD status may serve as a valuable component of the clinical profile in septic elderly patients. The observed relationships warrant further investigation in larger, longitudinal studies to determine their nature and to evaluate whether nutritional interventions could be beneficial for this vulnerable cohort.

## Data Availability

The original contributions presented in this study are included in this article/supplementary material, further inquiries can be directed to the corresponding authors.
